# Formulating an anesthetic plan using a list of high-risk pregnant women on cloud-based business communication tools: a case report

**DOI:** 10.1186/s40981-021-00430-2

**Published:** 2021-03-22

**Authors:** Junnosuke Kimura, Kento Kawamura, Manami Minoura, Ayako Hiramoto, Yoshifumi Suga, Shoko Okahara, Rie Inoue, Hiroyuki Sumikura

**Affiliations:** Department of Anesthesiology, Juntendo University Hospital, 3-1-3 Hongo Bunkyo-ku, Tokyo, 113-8431 Japan

**Keywords:** Cloud-based business communication tools, Antenatal anesthetic evaluation, List of high-risk pregnant women

## Abstract

**Background:**

We report a case in which a list of high-risk pregnant women on cloud-based business communication tools was useful in formulating an anesthetic plan for unscheduled cesarean section.

**Case presentation:**

A 37-year-old woman, who had been prescribed icosapentate for hypertriglyceridemia, received an antenatal anesthetic evaluation for possible cesarean delivery, and it was agreed that the anesthetic method for emergency cesarean section was general anesthesia if the surgery would take place within 7 days after the discontinuation of the drug, and regional anesthesia if it would take place any time later. Then this agreement was uploaded on the cloud-based business communication tools, and updated until she delivered her baby via unscheduled cesarean section.

**Conclusions:**

A cloud-based business communication tools was useful in formulating an anesthesia plan for a patient undergoing a cesarean delivery. However, more discussion would be needed to utilize it in security.

## Background

Cesarean delivery is often proposed as an emergency surgery even if it was initially planned as an elective one. Therefore, it is recommended that pregnant women with possible risk of cesarean section receive an antenatal anesthetic evaluation in advance [1, 2]. Especially pregnant women carrying some form of a risk (high-risk pregnant women) require additional special measures to be taken; hence, it has been encouraged to prepare a list of high-risk patients to share their information among the stuff of obstetric anesthesia team. However, in many cases, such lists are either filled out on a whiteboard in a staff room or stored on an isolated computer, making it difficult to be checked from outside the hospital. Here, we report a case in which a list of high-risk pregnant women on cloud-based business communication tools (BCT) was useful in formulating an anesthetic plan for unscheduled cesarean delivery.

## Case presentation

The patient was a 37-year-old woman (G3, P2), and written informed consent for publication was obtained. She has been taking oral prednisolone for controlling membranoproliferative nephropathy from the age of 19 years. Her past history included uneventful vaginal delivery at the age of 26 and cesarean delivery for preeclampsia at the age of 31. On this occasion, she was admitted to our hospital at 27 weeks and 3 days of gestation due to deterioration of kidney function and hypertension. On the day of admission, she received the first anesthetic evaluation, and her information was uploaded after excluding personally identifiable items in our list of high-risk pregnant women on the BCT.

At 30 weeks and 1 day of gestation, a stuff of the obstetric anesthesia team found that she had been taking icosapentate for hypertriglyceridemia through the regular anesthetic evaluation preparing for a possible cesarean delivery and discontinued its prescription after discussing with the relevant department. At that time, it was planned that anesthetic method for cesarean delivery was general (≤ 7 days) or regional (> 7 days after stopping icosapentate) according to the Japanese guideline [3]. Then this plan was recorded in her medical chart and updated in the BCT.

At 30 weeks and 4 days of gestation, because of progressing hypertension and deteriorating kidney function, cesarean section on 30w6d, after administration of betamethasone for two days to avoid infant respiratory distress syndrome, was scheduled. Although it was Saturday when her cesarean section was fixed, a resident of the obstetric anesthesia team visited the patient and explained general anesthesia for cesarean section. He also proposed transversus abdominis plane block for postoperative pain management and uploaded her acceptance on the BCT.

In Sunday morning, a senior anesthesiologist found the updated information on the BCT at home and left his comment that anesthetic plan should be reconsidered after checking the indication of peripheral nerve block for the patient receiving icosapentate before the surgery. After reading this comment, another resident, who was in charge on Sunday, confirmed that transversus abdominis plane block was relatively contraindicated in such situations. Therefore, he re-visited the patient to change the anesthetic plan for postoperative pain treatment from transversus abdominis plane block to intravenous patient-controlled analgesia. As she accepted this modification, the modified plan was updated on the BCT.

On the day of surgery (Monday), the patient started fasting from 12:00 AM and was later placed under general anesthesia to undergo cesarean section. The surgery was completed without any complications. Postoperative pain management was well-controlled by intravenous patient-controlled analgesia.

## Discussion

Here, we experienced an optimal anesthesia management according to the patient’s condition, wherein multiple anesthesiologists were able to discuss and formulate on the best anesthetic plan for a high-risk pregnant woman, even on a weekend, through sharing of her information on a cloud-based BCT.

Cesarean delivery often takes place as a form of emergency surgery, and anesthesiologists struggle to formulate the anesthetic plan. While it is beneficial for an anesthesiologist to have the antenatal anesthetic evaluation of a patient completed beforehand to avoid emergency situations, the patient’s condition changes dramatically even after such antenatal evaluation. In particular, for high-risk pregnant women, it is necessary to change the anesthetic plan in a flexible manner, according to the changes in her condition with time. Until recently, we had prepared a list of high-risk pregnant women and kept it in the isolated computer in the hospital to share their information, but had to access the medical charts in order to acquire the details of up-to-date information. For improving this situation, we started sharing the information of high-risk pregnant women with a cloud-based BCT service (Slack, Slack Technologies Inc. San Francisco, CA, USA) from October 2020.

By introducing this system, in-house residents were able to update the patient information at any time. Most importantly, it became possible for senior doctors not on site to check the latest status of the patient at a time convenient to them, from outside the hospital. As a result, it became possible to formulate the most suitable anesthetic plan for the patient, because the off-duty staff was able to check and revise the anesthesia plan made by an in-house resident.

To our best knowledge, we are not aware of any trouble using the BCT until today. However, one must be wary of protecting the personal information of patients. Although we have not obtained an official approval for sharing the patients’ data using this system from the ethical committee of our hospital, we believe that its security level is reliable as the BCT that we are using is a paid service and it has been adopted by major corporations. Even then, we are anonymizing the patient names and refraining from uploading any personal information about the patient other than the scheduled delivery date. We are also password-protecting any files that are shared between the relevant staff. Any member of the staff accessing the information from outside the hospital is being obliged to do so in an environment where there will be no leakage of information.

## Conclusion

Here, we have reported a case wherein cloud-based BCT proved useful in formulating an anesthesia plan for a patient undergoing a cesarean delivery. However, more discussion would be needed on sharing of patient information safely using BCT.

## Data Availability

Not applicable

## Abbreviation

BCT: Business communication tools
